# Prevalence of *Chlamydia trachomatis*, *Neisseria gonorrhoeae*, and *Ureaplasma urealyticum* infections in males and females of childbearing age in Chengdu, China

**DOI:** 10.3389/fcimb.2025.1566163

**Published:** 2025-04-28

**Authors:** Yuwei Li, Zhiyong Liao, Qin Wang, Weijun He, Yao Deng, Chenggui Liu

**Affiliations:** ^1^ Chengdu Women’s and Children’s Central Hospital, School of Medicine, University of Electronic Science and Technology of China, Chengdu, China; ^2^ Department of Clinical Laboratory, Chengdu Women’s and Children’s Central Hospital, School of Medicine, University of Electronic Science and Technology of China, Chengdu, China; ^3^ Department of Clinical Laboratory, Sichuan Province Orthopedic Hospital, Chengdu, China

**Keywords:** sexually transmitted infections, *Chlamydia trachomatis*, *Neisseria gonorrhoeae*, *Ureaplasma urealyticum*, childbearing age

## Abstract

**Background:**

Sexually transmitted infections (STIs) are a global public health issue, due to their high prevalence and potential impact on pregnancy outcome and fetal health. The objective of this study is to investigate the prevalence of STI-causative pathogens including *Chlamydia trachomatis* (CT), *Neisseria gonorrhoeae* (NG) and *Ureaplasma urealyticum* (UU) as well as the epidemiological characteristics of STIs among males and females of childbearing age in Chengdu.

**Methods:**

This retrospective cross-sectional study involved 15,055 participants of childbearing age including 7,235 males and 7,820 females. All specimens of participants were tested for CT, NG, and UU by nucleic acid amplification tests (NAATs) methods.

**Results:**

27.80% of the participants were infected with at least one of the three pathogens, with significantly higher overall prevalence in females (45.22%) than males (8.98%, *p*<0.001). Multivariate logistic regression analysis demonstrated that gender was independently associated with both CT positivity (females vs males, OR 2.276, 95% CI 1.724-3.005, *p*<0.001) and UU positivity (females vs males, OR 8.079, 95% CI 7.183-9.086, *p*<0.001). Single infections predominated in both males and females (males: 98.15%; females: 93.16%), while mixed infections were more frequent in females (6.84%) than in males (1.85%). CT prevalence was highest in both males and females aged 18-24, followed by aged 25–30 and 31-35. Among aged 18-24, the prevalence of UU in males and NG and UU in females were also higher. Compared to other age groups, mixed infections (CT+NG, CT+UU, NG+UU, CT+NG+UU) were also highest in females aged 18-24. Compared to other clinical diagnostic groups, The prevalence of CT, NG and mixed infection of CT+NG was highest in both males and females in the urogenital inflammation group (*p*<0.05).

**Conclusions:**

Among the population of childbearing age in Chengdu, China, the prevalence among females was significantly higher than that among males. Single infections predominated in both males and females, while mixed infections occurred more frequently in females. STIs were more prevalent in sexually active young people aged 18-35, especially in the 18–24 age group. CT and NG infections in both males and females may cause urogenital inflammation, and mixed infections of CT+NG further elevate the risk of inflammatory responses.

## Introduction

Sexually transmitted infections (STIs) are a major global public health problem that is still challenging, especially among adolescents and young adults, due to considerable morbidity, mortality and health expenditures, which have a profound impact on sexual and reproductive health (Zheng et al., 2022; Rodrigues et al., 2023; World Health Organization, 2024). According to the assessment of the World Health Organization (WHO), every day, more than one million curable STIs are acquired in the global population aged 15 to 49, and most of these infections are asymptomatic (World Health Organization, 2024). STIs in the urogenital tract can lead to urogenital symptoms and multiple adverse sexual and reproductive health outcomes, including infertility, cervical cancer, pregnancy complications and adverse birth and newborn outcomes, such as preterm birth, low birth weight, and perinatal death (Medina-Marino et al., 2022; Sameni et al., 2022; Gottlieb et al., 2024). In addition, STIs are manifested through stigmatization, which may result in psychological distress, social isolation, and economic consequences, seriously affecting quality of life (Griner et al., 2022; Gottlieb et al., 2024). More than 30 different pathogens such as bacteria, viruses and parasites can cause STIs through sexual activity (Gottlieb et al., 2014). Among them, Chlamydia trachomatis (CT), Neisseria gonorrhoeae (NG) and Ureaplasma urealyticum (UU) are three of the most common pathogens of STIs in the urogenital tracts (Grad et al., 2020; Liu et al., 2024).

CT is an obligate intracellular pathogen, and humans are its only natural host. CT infection in the urogenital tract is the most commonly diagnosed bacterial STI globally, occurring in the genitals (urethra or vagina/cervix) and characterized by urogenital inflammation, which will increase the risk of infertility, ectopic pregnancy and chronic pelvic pain (Witkin et al., 2017; Hocking et al., 2023). NG is a strict human parasitic bacterium that often exists in the white blood cells of purulent secretions in acute urethritis and vaginitis. It is a cause of pelvic inflammatory disease in females, which can lead to serious reproductive complications including tubal infertility, ectopic pregnancy, and chronic pelvic pain (Kirkcaldy et al., 2016; Młynarczyk-Bonikowska et al., 2020). UU is a small and fastidious pathogen with a circular double-stranded DNA genome but without a cell wall, which is not sensitive to detection by routine bacterial cultures and requires specialized media and growth conditions for optimal recovery (Cunningham et al., 2013; Oh et al., 2024). It is one of the most common pathogens causing urogenital infections and is related to complications like infertility, spontaneous abortion and other STIs (Ghorbanzadeh et al., 2020). Maternal UU infection may result in preterm birth, low birth weight and neonatal pneumonia (Liu et al., 2022).

There are various methods for detecting pathogens in the urogenital tract, including microscopy, culture, immunoassay, and nucleic acid amplification tests (NAATs) (Mallik et al., 2020; Caruso et al., 2021; Hopkins et al., 2023). Microscopy and culture are ideal methods for confirming the diagnosis of patients and identifying a reasonable treatment plan, and even culture is considered the gold standard for identifying pathogen infections (Abayasekara et al., 2017; Wolf et al., 2021). However, traditional selective culture methods for detecting CT, NG, and UU in clinical specimens have very low sensitivity (Serra-Pladevall et al., 2015; Liang et al., 2018) and microscopy of NG also has lower sensitivity (Shipitsyna et al., 2008). Moreover, culture requires a longer time with a more complex operation (Rudkjøbing et al., 2016; Caruso et al., 2021). Sometimes, picking colonies of pathogens and judging results of identification may cause false positives or false negatives due to subjective reasons of different testers (difficult to standardize visual judgments), which bring incorrect information for clinical diagnosis and epidemiological investigations.

Immunoassay to detect antigens or antibodies can also be used for the diagnosis of STIs. Antigen detection of pathogens (CT, NG) is more rapid and simpler than culture (Guy et al., 2017; Peng et al., 2020). However, antigen detection of pathogens (CT, NG) is not recommended as routine screening and diagnostic tests for STIs infections because of the suboptimal diagnostic accuracy (Meyer and Buder, 2020; Peng et al., 2020). Although serological assays for the presence of serum antibodies can be used to diagnose chronic infections and predict complications (Peng et al., 2020), these assays have some drawbacks in that they detect past exposure to the pathogen and have little value in screening for uncomplicated urogenital pathogen (CT) infection, and further testing is needed to confirm the presence of a current active infection (Meyer, 2016). In addition, whether detecting antigens or antibodies, there may be cross reactions leading to false positives, which limits the application of immunoassay for detecting pathogens of STIs (Samarawickrama et al., 2011; Rahman and Kaltenboeck, 2021).

The diagnosis of STIs has been significantly improved owing to remarkable progress in molecular biology technology. NAATs, which possess high sensitivity and specificity, have exhibited excellent performance. These tests have been extensively used in clinical laboratories for the detection of various pathogens (Güralp et al., 2019; Workowski et al., 2021; Tuddenham et al., 2022). In China, few large-scale studies have been published on the prevalence of CT, NG, and UU among males and females of childbearing age. In the present study, we employ the real-time polymerase chain reaction (RT-PCR) method to detect CT, NG, and UU in the urogenital specimens collected from males and females of childbearing age in Chengdu, China. The aim is to investigate the prevalence of these three STI pathogens and to explore the epidemiological characteristics of CT, NG, and UU within this specific area, thereby facilitating the development of more effective prevention and treatment strategies for urogenital tract STIs.

## Materials and methods

### Study participants

**Figure 1:**
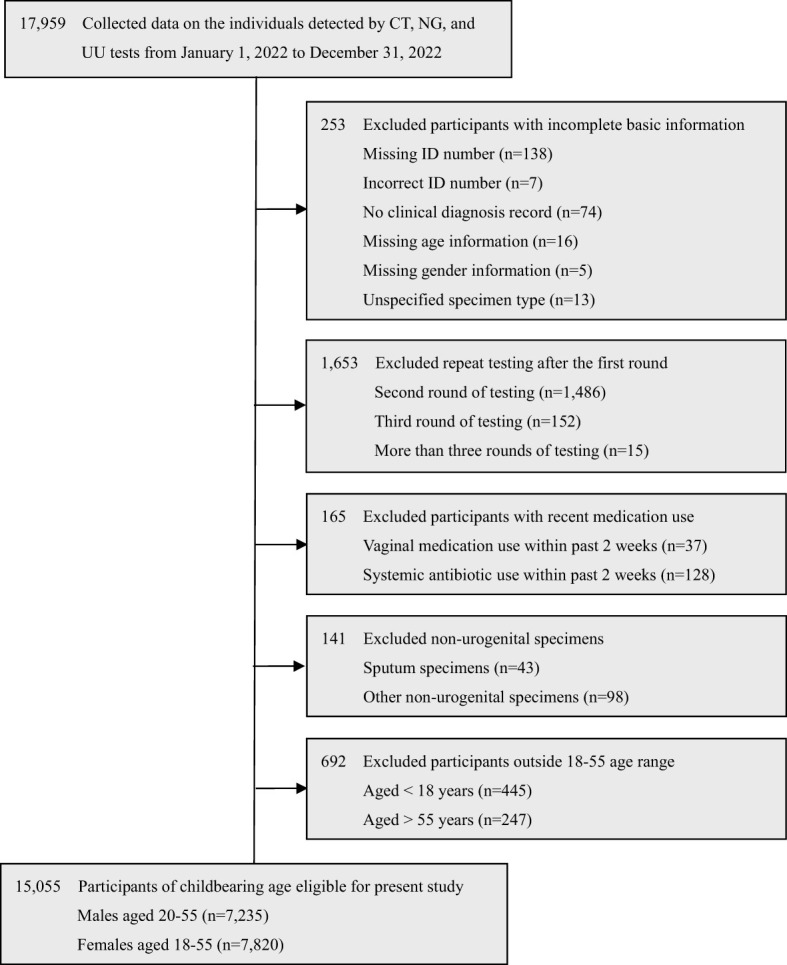
Selection of male and female participants within the childbearing age of 18-55.

The retrospective cross-sectional study was conducted from January 1, 2022, to December 31, 2022, at Chengdu Women’s and Children’s Central Hospital, School of Medicine, University of Electronic Science and Technology of China. A total of 17,959 individuals were tested for CT, NG, and UU tests. Among them, 15,055 eligible participants of childbearing age were selected in this study, including 7,235 males aged 20-55 (mean age: 31.85 ± 4.77) and 7,820 females aged 18-55 (mean age: 31.34 ± 5.28). The selection process is detailed in **Figure 1**.

The exclusion criteria were as follows: (a) Incomplete data: Participants lack some basic information, such as missing or incorrect ID number, no clinical diagnosis record, missing age or gender information, or unspecified specimen type. (b) Repeat testing: Participants who underwent repeat CT, NG, and UU testing after the first round. (c) Recent antimicrobial use: Participants who used vaginal medications or systemic antibiotics within the past 2 weeks. (d) Non-urogenital specimens: Participants whose specimens were not from the urogenital tract. (e) Age restrictions: Participants aged <18 or >55 years.

Among the 7,235 males screened, 7,088 specimens were obtained for CT testing, 1,835 for NG testing, and 7,235 for UU testing. Among the 7,820 females screened, 7,545 specimens were obtained for CT testing, 4,649 for NG testing, and 7,820 for UU testing. The total number of individuals tested, and the number of CT, NG, and UU tests performed among males and females of childbearing age in different age groups are presented in **Table 1**.

**Table 1:** Total number of individuals tested and the number of CT, NG, and UU tests performed among males and females of childbearing age in different age groups in Chengdu from January to December 2022.

Age	Males				Females			
	Total test (n)	CT test (n)	NG test (n)	UU test (n)	Total test (n)	CT test (n)	NG test (n)	UU test (n)
18–24 y	146	144	36	146	424	402	229	424
25–30 y	3008	2955	475	3008	3386	3282	1778	3386
31–35 y	2743	2685	785	2743	2590	2501	1617	2590
36–45 y	1029	998	388	1029	1272	1226	933	1272
46-55y	309	306	151	309	148	134	92	148

This study classified patients into 6 clinical diagnostic groups based on their different clinical diagnoses. When a patient has multiple clinical diagnoses, the first clinical diagnosis in the following order is selected: urogenital inflammation, history of adverse pregnancy and childbirth, infertility, pregnancy and fertility problems, health examination, and others.

1. Urogenital inflammations: (a) Males include urethritis, epididymitis, spermatocystitis, prostatitis and balanitis. (b) Females include urethritis, vaginitis, cervicitis and pelvic inflammatory diseases, which consist of endometritis and adnexitis.
2. History of adverse pregnancy and childbirth: Only for females, it includes biochemical pregnancy, spontaneous abortion, missed abortion, threatened premature delivery, ectopic pregnancy, placental abruption, premature rupture of membranes, and fetal growth arrest.
3. Infertility: includes primary infertility and secondary infertility.
4. Pregnancy and Fertility Problems: (a) Males include Azoospermia, Oligozoospermia, Asthenozoospermia, Teratozoospermia, Cryptozoospermia, Abnormal Sperm Syndrome, Absence of Vas Deferens, Sperm DNA Damage, Ejaculation Dysfunction, Retrograde Ejaculation, Erectile Dysfunction, Varicocele, Premature Ejaculation, Decreased Libido, Testicular Prolapse, etc. (b) Females include Polycystic Ovarian Syndrome, Abnormal Uterine Bleeding, Ovulation Disorder, Underdeveloped Follicles, Hypovaria, Ovarian Cysts, Adenomyosis, Uterine Fibroids, Endometriosis, Congenital Malformation of Fallopian Tubes, Immature Uterus, Unicornuate Uterus, Placental Thickening, Placenta Previa, Luteal Insufficiency, etc.
5. Health Examination: includes pre-marital health examination and pre-pregnancy health examination.
6. Others: such as Hashimoto’s Thyroiditis, Hypothyroidism, Insulin Resistance, HBV carriers, Mastitis, Breast Nodules, Vitamin D Deficiency, Psoriasis, Intrahepatic Cholestasis of Pregnancy, Hypertensive Disorders in Pregnancy, Thrombophilia, etc.

### Specimen collection

Urogenital swab sampling is carried out by clinicians or nurses following these procedures: (1) Urethral discharge specimen: Clean the urethral orifice of males or females with sterile saline. Then, use a sterile swab to enter 2–3 cm deep into the urethra, stay for a few seconds, rotate it firmly 1–2 turns, and remove the swab. (2) Cervical discharge specimen: Clean the cervix of females with sterile saline. Then, use a sterile swab to enter 1–2 cm deep into the cervix, stay for a few seconds, and remove the swab after 1–2 turns of vigorous rotation. The collected specimens are immediately placed in preservation solution. Transport workers transport them to the molecular biology laboratory within 2 hours and laboratory technicians store them in a specific refrigerator at 2-8°C until further processing. All specimens are completely tested within 24 hours.

### DNA extraction and the PCR assay

CT, NG, and UU in urogenital specimens were tested using RT-PCR (Shanghai ZJ Bio-Tech Co., Ltd., Shanghai, China).

**(1)** DNA extraction: According to the standard operating procedure (SOP), 1 mL of each specimen was centrifuged at 13,000 g for 5 min at room temperature. The supernatant was discarded (try not to touch the sediment as much as possible) and the sediment was washed twice with sterile saline. After the second washing, the sediment was added to 50 μL DNA extraction buffer (Shanghai ZJ Bio-Tech Co., Ltd., Shanghai, China) and thoroughly mixed as well as heated at 100°C for 10 min. Then, it was centrifuged again at 13,000 g for 5 min. The supernatant was collected and used as DNA extracts.

**(2)** PCR assay: A 40 μL reaction system for each test was prepared according to the manufacturer’s instructions. The PCR reaction solution and enzyme (Taq+UNG) in the reagent kit were taken out and melted at room temperature, mixed well, and centrifuged. 36 μL PCR reaction solution and 0.4 μL enzyme (Taq+UNG) were vigorously mixed for a few seconds and were centrifuged. Then, 36 μL of the mixture was taken out, placed into a PCR tube, and mixed with 4 μL of the previously prepared DNA extract. The reaction tube was covered with a cap. The amplification reaction was performed on a Tianlong Real-Time PCR System Gentier 96E (Xi’an Tianlong Science and Technology Co., Ltd, Xi’an, China) under the following conditions: 37°C for 2 min; 94°C for 2 min; 40 cycles of 93°C for 15 s; 60°C for 60 s. Positive, negative, and internal controls were included in each batch of testing.

### Statistical analysis

All analyses were performed with SPSS software version 19.0 (SPSS, Inc., Chicago, IL, USA). The prevalence of CT, NG, and UU was presented as percentages and analyzed using the Chi-Square test (*χ* ^2^). The associations between CT, NG, and UU positivity and variables, including gender, age, and clinical diagnoses were first evaluated using univariate logistic regression analysis. Variables with a *p*-value < 0.25 in the univariate analysis were considered potentially significant and were retained for further analysis. Subsequently, multivariate logistic regression analysis was conducted to evaluate the independent effects of these variables on CT, NG, and UU positivity. The Stepwise Forward Wald method was used to select the most significant variables for the final model, while controlling for potential confounding factors. Odds ratios (OR) and their 95% confidence intervals (95% CI) were calculated to quantify the strength and direction of the associations. This approach allowed for the identification of variables that independently contributed to the outcomes, providing a more robust understanding of the relationships between the predictors and CT, NG, and UU positivity. A *p*-value less than 0.05 was considered statistically significant.

## Results

### Prevalence of CT, NG, and UU infections in males and females of childbearing age

**Figure 2:**
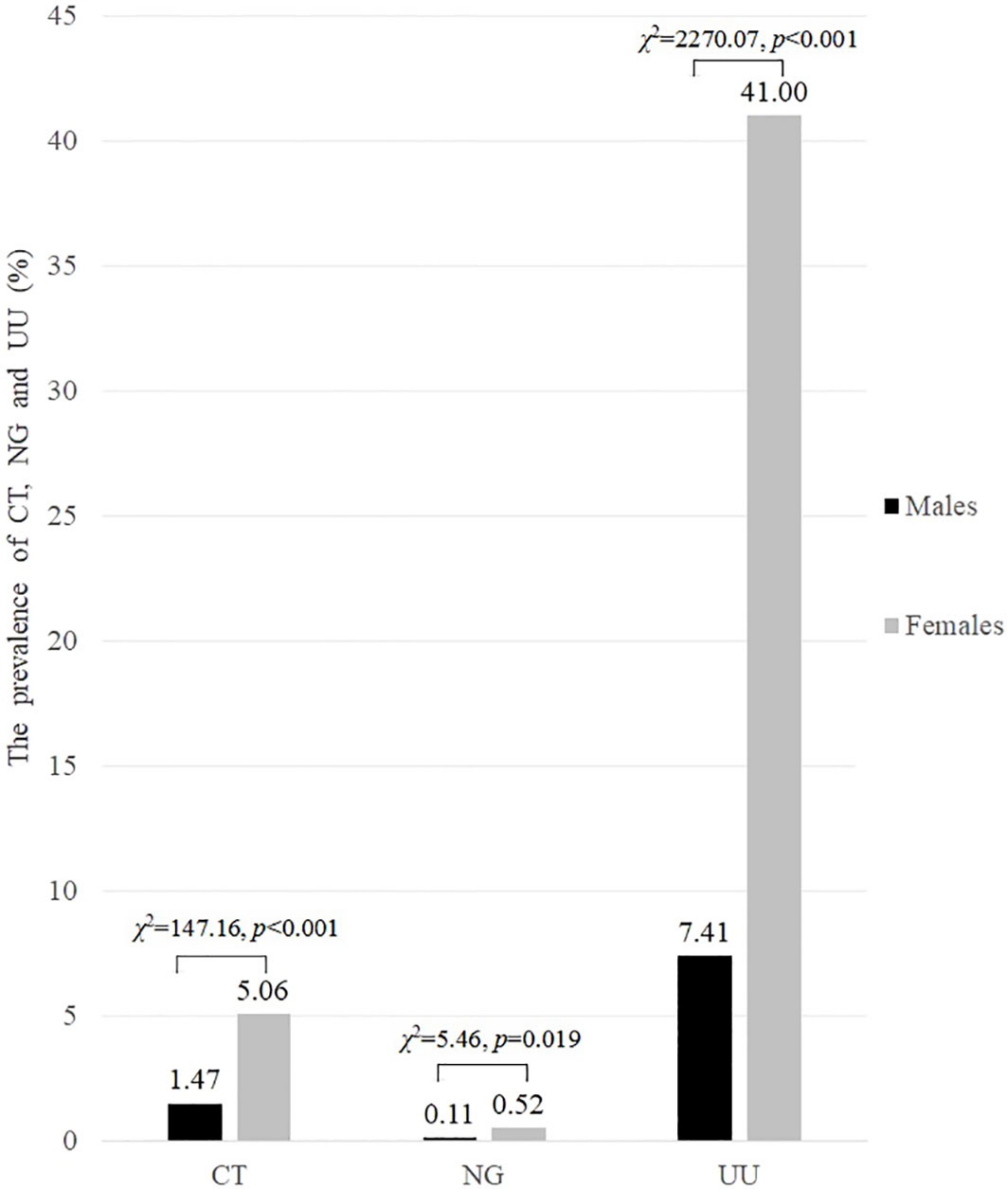
The prevalence of CT, NG and UU infections in both males and females of childbearing age (%).

Among 15,055 participants of childbearing age, 27.80% were infected with at least one of the three pathogens. The overall prevalence among females was significantly higher than that among males (45.22% vs 8.98%, *χ* ^2^ = 1432.94, *p* < 0.001). Among females, UU had the highest prevalence at 41.00%, followed by CT at 5.06%, while NG had the lowest prevalence at 0.52%. Similarly, among males, UU also had the highest prevalence at 7.41%, followed by CT at 1.47%, while NG had the lowest prevalence at 0.11%. The prevalence of CT, NG and UU in females was significantly higher than that in males (*p* < 0.05), as shown in **Figure 2**.

The proportion of participants with single infections of CT, NG, and UU was over 90% for both males and females (98.15% for males and 93.16% for females), while less than 7% had mixed infections (1.85% for males and 6.84% for females). Compared to males, females showed significantly higher prevalence of single infections of CT (3.11% vs 1.44%, *p* < 0.001) and UU (39.05% vs 7.39%, *p* < 0.001), as well as mixed infections of CT+UU (2.80% vs 0.16%, *p* < 0.001) and NG+UU (0.32% vs 0%, *p* = 0.015). However, there was no significant difference in the prevalence of NG single infection, CT+NG mixed infections, and CT+NG+UU mixed infections between females and males (*p* > 0.05). The comparison of the prevalence of single and mixed STIs of CT, NG, and UU between males and females is reported in **Table 2**.

**Table 2:** The prevalence of single and mixed STIs of CT, NG, and UU in both males and females.

STI types	Males		Females		*χ* ^2^	*p*-value
	n	Prevalence (%)	n	Prevalence (%)		
CT single infection	7,088	1.44	7,545	3.11	45.61	< 0.001
NG single infection	1,835	0.05	4,649	0.11	0.40	0.527
UU single infection	7,235	7.39	7,820	39.05	2074.62	< 0.001
CT+NG mixed infections	1,835	0.05	4,649	0.22	2.00	0.157
CT+UU mixed infections	7,088	0.16	7,545	2.80	170.66	< 0.001
NG+UU mixed infections	1,835	0	4,649	0.32	5.93	0.015
CT+NG+UU mixed infections	1,835	0	4,649	0.13	2.37	0.124

### Comparison of prevalence of CT, NG, and UU in different age groups

The prevalence of CT was highest in both males and females aged 18-24 (4.17% for males and 17.66% for females, respectively), followed by the age groups of 25–30 and 31-35, which were significantly higher than the age groups of 36–45 and 46-55 (*p* < 0.05). The prevalence of NG was significantly higher among females aged 18-24 (3.93%) and 46-55 (4.35%) than that among females in other age groups (*p* < 0.05). However, there was no significant difference in the prevalence of NG among males of different age groups (*p* = 0.820). The prevalence of UU was highest in females aged 18-24 (49.29%), significantly higher than that in other age groups (*p* = 0.002). Among males, the prevalence of UU was highest in those aged 18-24, 25-30, and 36-45; followed by the 31–35 age group; and the 46–55 age group had the lowest prevalence (*p* = 0.002). The comparison of the prevalence of CT, NG, and UU in different age groups for males and females is reported in **Table 3**.

**Table 3:** Comparison of prevalence of CT, NG, and UU in different age groups in males and females.

Age	Males			Females		
	CT prevalence (%)	NG prevalence (%)	UU prevalence (%)	CT prevalence (%)	NG prevalence (%)	UU prevalence (%)
18–24 y	4.17	0	8.22	17.66	3.93	49.29
25–30 y	1.83^a^	0	7.91	5.21 ^a^	0.39 ^a^	39.93 ^a^
31–35 y	1.45^a^	0.13	6.78	4.28 ^a^	0.25 ^a^	40.04 ^a^
36–45 y	0.50 ^a,b,d^	0.26	8.94^e^	2.53 ^abd^	0 ^a^	43.00 ^a^
46-55y	0 ^abd^	0	2.59 ^a,b,d,f^	1.49 ^abd^	4.35 ^c,e,g^	41.22 ^a^
*χ* ^2^	20.92	1.54	16.81	156.04	85.90	16.76
*p*-value	< 0.001	0.820	0.002	< 0.001	< 0.001	0.002

a Significantly decreased compared to 18–24 year group.

b Significantly decreased compared to 25–30 year group.

c Significantly increased compared to 25–30 year group.

d Significantly decreased compared to 31–35 year group.

e Significantly increased compared to 31–35 year group.

f Significantly decreased compared to 36–45 year group.

g Significantly increased compared to 36–45 year group.

### Comparison of prevalence of CT, NG, and UU in different clinical diagnoses

The prevalence of CT and NG was highest in both males and females in the urogenital inflammation group (18.52% for CT and 14.29% for NG in males, and 8.02% for CT and 1.07% for NG in females, respectively). This prevalence was significantly higher than that in other clinical diagnostic groups: history of adverse pregnancy and childbirth, infertility, pregnancy and fertility problems, health examination, and the others (*p* ≤ 0.001). For males, the second highest CT prevalence was in the pregnancy and fertility problems group (2.74%), and the third highest was in the infertility group (1.52%). For females, the second highest CT prevalence was in the infertility group (5.20%), and the third highest was in the pregnancy and fertility problems group (4.40%). However, whether males or females, there was no significant difference in the prevalence of UU among these six clinical diagnostic groups (*p* > 0.05). The comparison of the prevalence of CT, NG, and UU in different clinical diagnoses for both males and females is reported in **Table 4**.

**Table 4:** Comparison of prevalence of CT, NG, and UU in different clinical diagnoses in males and females.

Clinical diagnoses	Males			Females		
	CT prevalence (%)	NG prevalence (%)	UU prevalence (%)	CT prevalence (%)	NG prevalence (%)	UU prevalence (%)
Urogenital inflammation	18.52	14.29	6.90	8.02	1.07	42.06
History of adverse pregnancy and childbirth	/	/	/	1.15^a^	0.20 ^a^	39.60
Infertility	1.52^a,b^	0 ^a^	8.32	5.20^ab^	0 ^a^	42.25
Pregnancy and fertility problems	2.74^a,b^	0 ^a^	9.40	4.40 ^ab^	0.19 ^a^	42.26
Health examination	1.39^a^	0 ^a^	6.73	2.55^a,b,c,d^	0 ^a^	37.20
Others	1.13^a,b^	0 ^a^	6.76	3.97 ^a,b^	0 ^a^	38.68
*χ* ^2^	57.51	260.43	7.01	94.10	20.75	10.19
*p*-value	< 0.001	< 0.001	0.136	< 0.001	0.001	0.070

a Significantly decreased compared to urogenital inflammation group.

b Significantly increased compared to history of adverse pregnancy and childbirth group.

c Significantly decreased compared to infertility group.

d Significantly decreased compared to pregnancy and fertility problems group.

### Association between CT, NG, and UU mixed infections and age as well as clinical diagnoses

The prevalence of mixed infections of CT+NG, CT+UU, NG+UU, and CT+NG+UU in males and females was 0.05% and 0.22%, 0.16% and 2.80%, 0% and 0.32%, and 0% and 0.13% respectively. Females exhibited higher prevalence of mixed infections than males, with statistically significant differences in CT+UU (2.80% vs 0.16%, *p* < 0.001) and NG+UU (0.32% vs 0%, *p* = 0.015). Among females, the prevalence of mixed infections was highest in the 18–24 years group: 2.62% for CT+NG, 9.95% for CT+UU, 2.18% for NG+UU, and 1.31% for CT+NG+UU, all significantly higher than in other age groups (*p* < 0.001). Among these, CT+UU showed the highest prevalence. In contrast, among males, there were no significant differences in the prevalence of CT, NG, and UU mixed infections across all age groups (*p* > 0.05).

Compared to other clinical diagnostic groups, the urogenital inflammation group showed the highest prevalence of CT+NG mixed infection in both males and females (7.14% in males and 0.51% in females, both *p* < 0.05). Among females with urogenital inflammation, the prevalence of CT+UU, and NG+UU mixed infections was also the highest, at 4.30%, and 0.66%, respectively, significantly higher than that in the other clinical diagnostic groups (*p* < 0.05). However, there were no significant differences in the prevalence of CT+UU, NG+UU, and CT+NG+UU mixed infections in males as well as CT+NG+UU in females across all clinical diagnoses groups (*p* > 0.05). The association between CT, NG, and UU mixed infections and age is reported in **Table 5**, while that between CT, NG, and UU mixed infections and clinical diagnoses is reported in **Table 6**.

**Table 5:** Association between mixed infections and age in males and females.

Age	Males				Females			
	CT+NG prevalence (%)	CT+UU prevalence (%)	NG+UU prevalence (%)	CT+NG+UU prevalence (%)	CT+NG prevalence (%)	CT+UU prevalence (%)	NG+UU prevalence (%)	CT+NG+UU prevalence (%)
Total	0.05 (1/1,835)	0.16 (11/7,088)	0 (0/1,835)	0 (0/1,835)	0.22 (10/4,649)	2.80 (211/7,545)	0.32 (15/4,649)	0.13 (6/4,649)
18–24 y	0 (0/36)	0.69 (1/144)	0 (0/36)	0 (0/36)	2.62 (6/229)	9.95 (40/402)	2.18 (5/229)	1.31 (3/229)
25–30 y	0 (0/475)	0.14 (4/2,955)	0 (0/475)	0 (0/475)	0.17 (3/1,778) ^a^	2.93 (96/3,282) ^a^	0.34 (6/1,778) ^a^	0.17 (3/1,778) ^a^
31–35 y	0 (0/785)	0.19 (5/2,685)	0 (0/785)	0 (0/785)	0 (0/1,617) ^a^	2.20 (55/2,501) ^a^	0.12 (2/1,617) ^a^	0 (0/1,617) ^a^
36–45 y	0.26 (1/388)	0.10 (1/998)	0 (0/388)	0 (0/388)	0 (0/993) ^a^	1.63 (20/1,226) ^a,b^	0 (0/933) ^a^	0 (0/933) ^a^
46-55y	0 (0/151)	0 (0/306)	0 (0/151)	0 (0/151)	1.09 (1/92)	0 (0/134) ^a,b^	2.17 (2/92) ^c,d,e^	0 (0/92) ^a^
*χ* ^2^	3.73	3.61	/	/	70.64	89.14	39.48	28.41
*p*-value	0.444	0.461	/	/	< 0.001	< 0.001	< 0.001	< 0.001

a Significantly decreased compared to 18–24 year group.

b Significantly decreased compared to 25–30 year group.

c Significantly increased compared to 25–30 year group.

d Significantly increased compared to 31–35 year group.

e Significantly increased compared to 36–45 year group.

**Table 6:** Association between mixed infections and clinical diagnoses in males and females.

Clinical diagnoses	Males				Females			
	CT+NG prevalence (%)	CT+UU prevalence (%)	NG+UU prevalence (%)	CT+NG+UU prevalence (%)	CT+NG prevalence (%)	CT+UU prevalence (%)	NG+UU prevalence (%)	CT+NG+UU prevalence (%)
Total	0.05 (1/1,835)	0.16 (11/7,088)	0 (0/1,835)	0 (0/1,835)	0.22 (10/4,649)	2.80 (211/7,545)	0.32 (15/4,649)	0.13 (6/4,649)
Urogenital inflammation	7.14 (1/14)	0 (0/27)	0 (0/14)	0 (0/14)	0.51 (10/1,963)	4.30 (111/2,580)	0.66 (13/1,963)	0.31 (6/1,963)
History of adverse pregnancy and childbirth	/	/	/	/	0 (0/510)	0.96 (10/1045) ^a^	0 (0/510)	0 (0/510)
Infertility	0 (0/1,425) ^a^	0.11 (3/2,764)	0 (0/1,425)	0 (0/1,425)	0 (0/769) ^a^	2.55 (27/1,058) ^ab^	0 (0/769) ^a^	0 (0/769)
Pregnancy and fertility problems	0 (0/138) ^a^	0 (0/146)	0 (0/138)	0 (0/138)	0 (0/1,036) ^a^	2.51 (36/1,432) ^ab^	0.19 (2/1,036)	0 (0/1,036)
Health examination	0 (0/84) ^a^	0.22 (5/2,297)	0 (0/84)	0 (0/84)	0 (0/95)	1.45 (12/825) ^a^	0 (0/95)	0 (0/95)
Others	0 (0/174) ^a^	0.16 (3/1854)	0 (0/174)	0 (0/174)	0 (0/276)	2.48 (15/605) ^ab^	0 (0/276)	0 (0/276)
*χ* ^2^	130.14	1.24	/	/	13.17	40.87	12.92	8.22
*p*-value	< 0.001	0.871	/	/	0.018	< 0.001	0.024	0.144

a Significantly decreased compared to urogenital inflammation group.

b Significantly increased compared to history of adverse pregnancy and childbirth group.

### Associations between CT, NG and UU positivity and variables of gender, age and clinical diagnoses by univariate logistic regression analysis

Univariate logistic regression analysis revealed that CT positivity was significantly associated with gender (females vs males, OR 3.581, 95% CI 2.876-4.459), age (25–30 years, 31–35 years, 36–45 years, and 46–55 years, all compared to the 18–24 years group), and clinical diagnoses (history of adverse pregnancy and childbirth, infertility, pregnancy and fertility problems, health examination, and others, all compared to the urogenital inflammation group) (all *p* < 0.001). NG positivity was significantly associated with gender (females vs males, OR 4.756, 95% CI 1.123-20.144), age (25–30 years, 31–35 years, and 36–45 years, all compared to the 18–24 years group), and clinical diagnoses (pregnancy and fertility problems compared to the urogenital inflammation group) (all *p* < 0.05). However, NG positivity was not significantly associated with the 46–55 years age group (*p* = 0.222), nor with the history of adverse pregnancy and childbirth group (*p* = 0.080), infertility group (*p* = 0.984), health examination group (*p* = 0.996), or others group (*p* = 0.993). UU positivity was significantly associated with gender (females vs males, OR 8.684, 95% CI 7.867-9.587), age (25–30 years, 31–35 years, 36–45 years, and 46–55 years, all compared to the 18–24 years group), and clinical diagnoses (infertility, health examination, and others, all compared to the urogenital inflammation group) (all *p* < 0.001). However, UU positivity was not significantly associated with the history of adverse pregnancy and childbirth group (*p* = 0.239), pregnancy and fertility problems group (*p* = 0.109). The associations between CT, NG, and UU positivity and variables of gender, age, and clinical diagnoses in childbearing age populations are reported in **Table 7**.

**Table 7:** Univariate logistic regression analysis of the association between CT, NG and UU positivity and variables of gender, age and clinical diagnoses in childbearing age populations.

	CT				NG				UU			
Variables	Odds ratio	95% CI	Wald	*p*-value	Odds ratio	95% CI	Wald	*p*-value	Odds ratio	95% CI	Wald	*p*-value
Gender	3.581	2.876-4.459	130.031	< 0.001	4.756	1.123-20.144	4.483	0.034	8.684	7.867-9.587	1836.817	< 0.001
Age												
18–24 y	1	−	−	−	1	−	−	−	1	−	−	−
25–30 y	0.228	0.173-0.300	110.811	< 0.001	0.089	0.033-0.240	22.728	< 0.001	0.523	0.438-0.624	51.165	< 0.001
31–35 y	0.176	0.132-0.236	135.760	< 0.001	0.059	0.020-0.178	25.293	< 0.001	0.470	0.392-0.563	67.486	< 0.001
36–45 y	0.100	0.067-0.151	122.064	< 0.001	0.022	0.003-0.171	13.198	< 0.001	0.607	0.501-0.735	26.051	< 0.001
46-55y	0.028	0.007-0.114	24.802	< 0.001	0.476	0.145-1.566	1.492	0.222	0.281	0.207-0.382	65.938	< 0.001
Clinical diagnoses												
Urogenital inflammation	1	−	−	−	1	−	−	−	1	−	−	−
History of adverse pregnancy and childbirth	0.131	0.073-0.236	46.111	<0.001	0.167	0.022-1.239	3.064	0.080	0.917	0.793-1.059	1.386	0.239
Infertility	0.294	0.230-0.376	95.282	< 0.001	0.000	0.000-/	0.000	0.984	0.301	0.269-0.336	444.037	< 0.001
Pregnancy and fertility problems	0.501	0.378-0.664	23.061	< 0.001	0.145	0.034-0.616	6.845	0.009	0.903	0.797-1.023	2.565	0.109
Health examination	0.195	0.144-0.265	109.782	< 0.001	0.000	0.000-/	0.000	0.996	0.241	0.213-0.273	506.718	< 0.001
Others	0.211	0.152-0.292	87.387	< 0.001	0.000	0.000-/	0.000	0.993	0.243	0.212-0.277	434.068	< 0.001

### Results of stepwise multivariate logistic regression analysis (stepwise forward wald)

After adjusting for potential confounders, the results of stepwise multivariate logistic regression analysis are shown in **Table 8**. Gender was independently associated with both CT positivity (females vs males, OR 2.276, 95% CI 1.724-3.005, *p* < 0.001) and UU positivity (females vs males, OR 8.079, 95% CI 7.183-9.086, *p* < 0.001). Compared to the 18–24 years group, all other age groups showed significantly lower risks of CT positivity (all ORs < 1, *p* < 0.001). Similarly, for UU positivity, all other age groups also showed significantly lower risks (all ORs < 1, *p* < 0.05). For NG positivity, the 25–30 years, 31–35 years, and 36–45 years age groups had significantly lower risks (all ORs < 1, *p* < 0.001), while no significant association was observed for the 46–55 years group (*p* = 0.632). Compared to the urogenital inflammation group, all other clinical diagnostic groups showed significantly lower risks of CT positivity (all ORs < 1, *p* < 0.001). For NG positivity, the pregnancy and fertility problems group showed significantly lower risks (OR 0.148, 95% CI 0.035-0.630, *p* = 0.010), while no significant associations were observed for the history of adverse pregnancy and childbirth, infertility, health examination, and others groups (all *p* > 0.05). For UU positivity, the health examination, and others groups showed significantly lower risks (both ORs < 1, *p* < 0.05), while no significant associations were observed for the history of adverse pregnancy and childbirth, infertility, and pregnancy and fertility problems groups (all *p* > 0.05).

**Table 8:** Multivariate logistic regression (Stepwise Forward Wald) for CT, NG and UU positivity in 15,055 participants of childbearing age aged 18-55.

	CT				NG				UU			
Variables	Odds ratio	95% CI	Wald	*p*-value	Odds ratio	95% CI	Wald	*p*-value	Odds ratio	95% CI	Wald	*p*-value
Gender	2.276	1.724-3.005	33.706	< 0.001	/	/	/	/	8.079	7.183-9.086	1214.200	< 0.001
Age												
18–24 y	1	−	−	−	1	−	−	−	1	−	−	−
25–30 y	0.310	0.233-0.412	64.995	< 0.001	0.116	0.042-0.317	17.618	< 0.001	0.731	0.604-0.884	10.385	0.001
31–35 y	0.218	0.161-0.294	98.960	< 0.001	0.077	0.025-0.233	20.562	< 0.001	0.693	0.571-0.840	13.895	< 0.001
36–45 y	0.103	0.068-0.157	114.861	< 0.001	0.025	0.003-0.196	12.242	< 0.001	0.799	0.651-0.982	4.551	0.033
46-55y	0.034	0.008-0.141	21.875	< 0.001	0.743	0.220-2.506	0.230	0.632	0.541	0.388-0.753	13.240	< 0.001
Clinical diagnoses												
Urogenital inflammation	1	−	−	−	1	−	−	−	1	−	−	−
History of adverse pregnancy and childbirth	0.115	0.064-0.206	52.023	< 0.001	0.187	0.025-1.408	2.649	0.104	0.902	0.779-1.044	1.925	0.165
Infertility	0.490	0.368-0.653	23.870	< 0.001	0.000	0.000-/	0.000	0.984	1.017	0.894-1.157	0.066	0.798
Pregnancy and fertility problems	0.494	0.371-0.658	23.289	< 0.001	0.148	0.035-0.630	6.675	0.010	1.011	0.890-1.148	0.026	0.871
Health examination	0.297	0.211-0.419	47.955	< 0.001	0.000	0.000-/	0.000	0.995	0.819	0.710-0.944	7.556	0.006
Others	0.342	0.237-0.492	33.338	< 0.001	0.000	0.000-/	0.000	0.993	0.850	0.731-0.990	4.391	0.036

According to the results of the univariate analysis, three variables consisting of gender, age (5 groups: 18–24 years, 25–30 years, 31–35 years, 36–45 years and 46–55 years), and clinical diagnoses (6 groups: urogenital inflammation, history of adverse pregnancy and childbirth, infertility, pregnancy and fertility problems, health examination and others) were included in the initial model. Among these, gender was excluded during the multivariate logistic regression analysis using the Stepwise Forward Wald method.

## Discussion

STIs are among the most common infectious diseases and affect the health and lives of hundreds of millions of people worldwide each year (Sun et al., 2021). Screening high-risk populations will significantly improve the prevention and treatment of STIs (Gannon-Loew and Holland-Hall, 2020). Moreover, conducting comprehensive detection on males and females of childbearing age can not only promptly identify those with STIs, especially asymptomatic carriers, thereby helping us provide rapid intervention and treatment to those infected and improving the situation of conjugal life, but also contribute to preventing the occurrence of adverse birth and newborn outcomes.

In the present study, more than a quarter (27.80%) of the participants were infected with at least one of the three pathogens. The overall prevalence among females was significantly higher than that among males (45.22% vs 8.98%, *p* < 0.001). Additionally, the prevalence of each of the three pathogens was also significantly higher in females than in males. Specifically, the prevalence of CT, NG, and UU was 5.06% vs 1.47%, 0.52% vs 0.11%, and 41.00% vs 7.41% (all *p* < 0.05), respectively. After adjusting for potential confounders, multivariate logistic regression analysis demonstrated that gender was independently associated with both CT positivity (females vs males, OR 2.276, 95% CI 1.724-3.005, *p* < 0.001) and UU positivity (females vs males, OR 8.079, 95% CI 7.183-9.086, *p* < 0.001).

The proportion of participants with single infections of CT, NG, and UU was over 90% for both males and females (98.15% for males and 93.16% for females), while less than 7% had mixed infections (1.85% for males and 6.84% for females). The prevalence of mixed infections of CT+NG, CT+UU, NG+UU, and CT+NG+UU in males and females was 0.05% and 0.22%, 0.16% and 2.80%, 0% and 0.32%, and 0% and 0.13% respectively. Compared to males, females showed significantly higher prevalence of single infections of CT (3.11% vs 1.44%, *p* < 0.001) and UU (39.05% vs 7.39%, *p* < 0.001), as well as mixed infections of CT+UU (2.80% vs 0.16%, *p* < 0.001) and NG+UU (0.32% vs 0%, *p* = 0.015).

These differences may be attributed to differences in urogenital anatomy between females and males. Compared to males, the vaginal mucosa of females is thin, delicate and easily penetrated by pathogens, making their urogenital system more exposed and vulnerable to STIs (Van Gerwen et al., 2022). Furthermore, in heterosexual sexual intercourse, females are typically the receptive party, which may be exposed to pathogens over a relatively larger area and for a longer duration. For example, during unprotected sex, pathogens in semen are more likely to stay in the female vagina, cervix and other sites and cause infections. In addition, during menstruation or pregnancy, changes in physiology and immunity will increase the risk of being infected by pathogens. During menstruation, the cervical is slightly open, making it easier for pathogens to enter the uterus and fallopian tubes. While during pregnancy, increased vaginal secretions and altered pH values may facilitate the growth and transmission of certain pathogens.

In a descriptive, observational, cross-sectional study in Cuenca, Ecuador by Abad et al (Abad et al., 2022), PCR and flow hybridization were used to test for CT, NG and UU in cervical specimens from 102 asymptomatic females of childbearing age ranging from 18 to 45 years. The results showed that among these pathogens, the prevalence was highest in UU (48.04%), followed by CT (2.94%), and lowest in NG (0%). In another large-scale and cross-sectional study conducted in Taizhou, China by Cai et al (Cai et al., 2020), RT-PCR was used to test CT, NG and UU in 13,303 vaginal specimens collected from females with an average age of 32.30 years (ranging from 13 to 89 years). This study revealed that the prevalence was highest in UU (62.04%), followed by CT (10.20%), and lowest in NG (4.09%). Our findings indicate that among these three STI pathogens, whether males or females, UU consistently exhibited the highest prevalence, followed by CT, while NG had the lowest prevalence. When focusing on females, the prevalence of CT, NG, and UU was 5.06%, 0.52%, and 41.00%, respectively, which was lower than that reported by Cai et al. but closer to the findings of Abad et al.

Numerous studies have demonstrated that the prevalence of STIs such as CT, NG, and UU are related to the age of patients (Naidoo et al., 2014; Cai et al., 2020; Park et al., 2020; Lu et al., 2023). In this study, the prevalence of CT was highest in both males and females aged 18-24 (4.17% for males and 17.66% for females, respectively), followed by the age groups of 25–30 and 31-35, with significantly higher prevalence than the age groups of 36–45 and 46-55. Among females, the prevalence of NG in the 18–24 age group was 3.93%, significantly higher than in the age groups of 25-30, 31-35, and 36-45. Similarly, the prevalence of UU among females aged 18–24 was highest (49.29%), significantly higher than in other age groups. Meanwhile, among males, the prevalence of UU in the age groups of 18-24, 25-30, and 31–35 was 8.22%, 7.91%, and 6.78%, respectively, all higher than in the 46–55 age group (2.59%).

Moreover, among females, mixed infections were also primarily concentrated in the 18–24 age group. The prevalence of mixed infections of CT+NG, CT+UU, NG+UU, and CT+NG+UU was highest in the 18–24 age group, at 2.62%, 9.95%, 2.18%, and 1.31%, respectively, significantly higher than in other age groups (*p* < 0.001). Among these, CT+UU showed the highest prevalence. After adjusting for potential confounders, multivariate logistic regression analysis demonstrated that, compared to the 18–24 years group, all other age groups showed significantly lower risks of CT positivity (all ORs < 1, *p* < 0.001). Similarly, for UU positivity, all other age groups also showed significantly lower risks (all ORs < 1, *p* < 0.05). For NG positivity, the 25–30 years, 31–35 years, and 36–45 years age groups had significantly lower risks (all ORs < 1, *p* < 0.001), while no significant association was observed for the 46–55 years group (*p* = 0.632).

Our research further confirms that STIs were more prevalent in sexually active young people aged 18-35, especially in the 18–24 age group which had a higher prevalence of CT and UU in males, and a higher prevalence of CT, NG and UU in females. The higher prevalence among young people can be attributed to several factors. Firstly, young people are sexually active, and some of them have multiple sexual partners or a high frequency of sexual intercourse. Secondly, young people are less likely to use condoms correctly during sexual intercourse compared with older people. Thirdly, the outward migration of the cervical columnar epithelium in young females increases the vulnerability of the cervical mucosa to pathogen invasion. Fourthly, young people lack specific immunity to pathogens during the initial stage of sexual life when they are first infected. In addition, some young people lack knowledge about sexual health, including the transmission routes, symptoms, and risks of STIs, which makes it difficult for them to protect themselves.

In addition to the relatively high prevalence of NG among younger females aged 18-24, this study found that the prevalence of NG among older females aged 46–55 was high, and it was significantly higher than that in the middle-aged groups of 25-30, 31–35 and 36-45 (*p* < 0.05). Our research results are consistent with those reported by Abad et al (Cai et al., 2020), who reported that the prevalence of NG was relatively high in younger age groups (< 25 years), lower in middle-aged groups, and females over 40 years old had the highest susceptibility to NG infection. The significantly increased prevalence of NG among females aged 46–55 may be related to changes in estrogen levels and having new sexual partners. For one thing, some females may experience a decrease in estrogen levels after menopause, which may result in thinning and drying of the vaginal mucosa, an increase in pH value, and a decrease in lactobacillus in the vagina. Consequently, these changes can lower the natural defense ability of the vagina and thus make it easier for NG to invade and reproduce. For another, some females in this age group may have new sexual partners. If the sexual partner is infected with NG and unprotected sex occurs, the risk of NG infection will increase.

Studies have reported significant associations of CT and NG infections with urogenital inflammation (Garrett et al., 2021; Wall et al., 2021). In specimens of male urethral discharge, NG and CT are the most common pathogens of STIs (Rietmeijer et al., 2018). For females, CT and NG are also the predominant pathogens causing urogenital inflammations such as cervicitis and pelvic inflammatory disease, which involves the uterus, fallopian tubes, ovaries, and/or pelvic peritoneum (Shroff, 2023). Our results demonstrated that the prevalence of CT and NG was highest in both males and females in the urogenital inflammation group (18.52% for CT and 14.29% for NG in males, and 8.02% for CT and 1.07% for NG in females, respectively), which was significantly higher than that in other clinical diagnostic groups (*p* ≤ 0.001). Furthermore, the prevalence of CT+NG mixed infections in both males and females in the urogenital inflammation group was the highest at 7.14% and 0.51%, respectively, which was also significantly higher than that in other clinical diagnostic groups (*p* < 0.05). In addition, among females with urogenital inflammation, the prevalence of CT+UU, and NG+UU mixed infections was also the highest, at 4.30%, and 0.66%, respectively, significantly higher than that in the other clinical diagnostic groups (*p* < 0.05). However, there were no significant differences in the prevalence of CT+UU, NG+UU, and CT+NG+UU mixed infections in males as well as CT+NG+UU in females across all clinical diagnoses groups (p > 0.05).

Multivariate logistic regression analysis showed that compared to the urogenital inflammation group, all other clinical diagnostic groups showed significantly lower risks of CT positivity (all ORs < 1, *p* < 0.001). For NG positivity, the pregnancy and fertility problems group showed significantly lower risks (OR 0.148, 95% CI 0.035-0.630, *p* = 0.010). For UU positivity, the health examination, and other groups showed significantly lower risks (both ORs < 1, *p* < 0.05). These findings suggest that CT and NG infections in both males and females may cause urogenital inflammation, and mixed infections of CT+NG further elevate the risk of inflammatory responses. The synergistic effects of CT and NG pathogens may promote the occurrence and development of urogenital inflammation. To address this issue, comprehensive measures should be implemented, including strengthening sexual health education, providing policy support, promoting multi-pathogen joint screening, and actively intervening and treating infections. These efforts should focus on the population of childbearing age, particularly sexually active young people, to reduce the prevalence of STIs and improve public health outcomes.

### Limitations

Firstly, this study was conducted at a single site, resulting in a relatively limited specimen source. In the future, it is necessary to conduct multi-center and large-scale research to improve the reliability of results and the universality of conclusions. Secondly, since retrospective research data rely on existing records, it is challenging to comprehensively control potential confounding factors. Therefore, a prospective study is needed to better track variable changes and ensure the reliability of the results. Thirdly, the present study focused only on the prevalence of CT, NG, and UU among males and females of childbearing age and did not examine the impacts of infections caused by these three pathogens on pregnant women and fetuses. In the future, we plan to investigate the impacts of CT, NG, and UU infections on pregnant women and their fetuses, with the aim of disclosing their interrelationships and underlying mechanisms, thereby providing more valuable evidence to safeguard maternal and infant health. Fourthly, the logistic regression analysis only included variables of gender, age, and clinical diagnosis. In future research, we plan to collect more variables, including educational background, occupation, number of sexual partners, frequency of sexual intercourse, individual immune status, cultural attitudes, level of sex education, and lifestyle habits (e.g., smoking, drinking), and to analyze the associations between STI prevalence and these variables, and explore the impacts of CT, NG, and UU infections on pregnant women and their fetuses, with the aim of elucidating their interrelationships and underlying mechanisms, thereby providing more valuable evidence to safeguard maternal and infant health.

## Conclusions

In the present study, more than a quarter (27.80%) of the population of childbearing age were infected with at least one of the three pathogens, with UU being the most common pathogen in both male and female groups. The overall prevalence among females was significantly higher than that among males (45.22% vs 8.98%, *p* < 0.001), indicating that females were more susceptible than males. Moreover, multivariate logistic regression analysis demonstrated that gender was independently associated with both CT positivity (females vs males, OR 2.276, 95% CI 1.724-3.005, *p* < 0.001) and UU positivity (females vs males, OR 8.079, 95% CI 7.183-9.086, *p* < 0.001). The single infection patterns were more common in both male and female groups, with a single infection prevalence of 98.15% in males and 93.16% in females. In contrast, mixed infections were significantly more prevalent in females (6.84%) than in males (1.85%), particularly CT+UU mixed infections which demonstrated marked female predominance. STIs were more prevalent among the sexually active young people aged 18-35, especially in the 18–24 age group. The urogenital inflammation group demonstrated significantly higher prevalence of CT, NG and their mixed infections (CT+NG) compared to other clinical diagnostic groups in both males and females, indicating these pathogens may play a role in urogenital inflammation, with CT+NG mixed infections potentially exacerbating local inflammatory responses.

## Data Availability

The raw data supporting the conclusions of this article will be made available by the authors, without undue reservation.
