# Correction: Prevalence of *Chlamydia trachomatis*, *Neisseria gonorrhoeae*, and *Ureaplasma urealyticum* infections in males and females of childbearing age in Chengdu, China

**DOI:** 10.3389/fcimb.2025.1662536

**Published:** 2025-08-01

**Authors:** Yuwei Li, Zhiyong Liao, Qin Wang, Weijun He, Yao Deng, Chenggui Liu

**Affiliations:** ^1^ Chengdu Women’s and Children’s Central Hospital, School of Medicine, University of Electronic Science and Technology of China, Chengdu, China; ^2^ Department of Clinical Laboratory, Chengdu Women’s and Children’s Central Hospital, School of Medicine, University of Electronic Science and Technology of China, Chengdu, China; ^3^ Department of Clinical Laboratory, Sichuan Province Orthopedic Hospital, Chengdu, China

**Keywords:** sexually transmitted infections, *Chlamydia trachomatis*, *Neisseria gonorrhoeae*, *Ureaplasma urealyticum*, childbearing age

In the published article, there was an error in affiliation 1. Instead of “School of Medicine, University of Electronic Science and Technology of China, Chengdu, China”, it should be “Chengdu Women’s and Children’s Central Hospital, School of Medicine, University of Electronic Science and Technology of China, Chengdu, China”.

The authors apologize for this error and state that this does not change the scientific conclusions of the article in any way. The original article has been updated.

